# PATIENT PRIORITIES CARE (PPC) IN HISPANIC PATIENTS WITH DEMENTIA

**DOI:** 10.1093/geroni/igae098.2638

**Published:** 2024-12-31

**Authors:** Alejandra Mera, Emily Peel, Aanand Naik, Rafael Samper-Ternent

**Affiliations:** The University of Texas Medical Branch, Houston, Texas, United States; McGovern Medical School, Houston, Texas, United States; The University of Texas Health Science Center, Houston, Texas, United States; The University of Texas Health Houston, Houston, Texas, United States

## Abstract

Hispanics are the fastest growing underrepresented population in the U.S and have a higher risk of dementia compared to non-Hispanic whites. Lack of trust, limited health care access, and lack of culturally adapted interventions contribute to health disparities in this population group. Patient Priorities Care (PPC) is an evidenced-based approach that improves health outcomes in patients with multimorbidity. PPC guides patients, caregivers, and their providers to set realistic health care goals based on the patient’s values. Little is known about the benefits of PPC for Hispanics with multimorbidity and dementia. We designed and implemented a pilot feasibility study to adapt the PPC approach for Hispanics with multimorbidity and dementia (PPC-HD study). The PPC approach consists of a Priorities Setting Session with the patient, and caregiver, followed by a Care Alignment session with the patient’s clinician. In our sample of 20 Hispanics with dementia and multimorbidity, 85% of the participant care plans include patients’ preferences indicating the willingness of clinicians to use the PPC approach. Eighty-three percent of participants find PPC a rewarding experience with 67% reporting satisfaction in the availability of Spanish-speaking research staff, 61% reporting satisfaction in discussing healthcare preferences, and 56% reporting satisfaction with knowing patients’ preferences for future healthcare decisions. PPC is a feasible approach in this population group. Most clinicians used the patients’ preferences and included them in their care plans. Access to bilingual staff and delivery of the intervention in Spanish was highly valued by participants and caregivers. Participation in PPC was deemed very valuable.

